# The Effect of Fat Content and Fatty Acids Composition on Color and Textural Properties of Butter

**DOI:** 10.3390/molecules26154565

**Published:** 2021-07-28

**Authors:** Sergiu Pădureţ

**Affiliations:** Faculty of Food Engineering, Stefan cel Mare University of Suceava, 13 University Str., 720229 Suceava, Romania; sergiu.paduret@fia.usv.ro

**Keywords:** butter, texture, fatty acids, color

## Abstract

The textural properties of butter are influenced by its fat content and implicitly by the fatty acids composition. The impact of butter’s chemical composition variation was studied in accordance with texture and color properties. From 37 fatty acids examined, only 18 were quantified in the analyzed butter fat samples, and approximately 69.120% were saturated, 25.482% were monounsaturated, and 5.301% were polyunsaturated. The butter samples’ viscosity ranged between 0.24 and 2.12 N, while the adhesiveness ranged between 0.286 to 18.19 N·mm. The principal component analysis (PCA) separated the butter samples based on texture parameters, fatty acids concentration, and fat content, which were in contrast with water content. Of the measured color parameters, the yellowness b* color parameter is a relevant indicator that differentiated the analyzed sample into seven statistical groups; the ANOVA statistics highlighted this difference at a level of *p* < 0.001.

## 1. Introduction

Butter represents a water-in-oil emulsion, being one of the oldest milk products [1]; its origin is unknown, but it probably dates back to the prehistoric stages of animal husbandry. In total, about one-third of the world’s milk production is destined for butter production [2]. Recently emulsions with smaller spherical droplet diameters (<100 nm) have increased interest due to their novel physicochemical properties [3]. According to Sikorski and Kolakowska (2010), in butter structure, water drops are smaller than 10 µm in diameter and covered by a shell of fat crystals, which separates the water drops from each other and prevents the coalescing process [4].

Food emulsions show significant dissimilarities in terms of physicochemical and organoleptic characteristics such as appearance, textural properties, flavor, taste, and shelf life [5].

Butter is a dairy product made exclusively through the churning process of the pasteurized cream, which has been separated from milk (generally cow’s milk), the excess water (buttermilk) being removed. The butter flavor is given by the diacetyl, other substances such as butyric, propionic and formic acid, acetaldehyde acetoin having a smaller contribution [6]. In addition to its high-fat content, butter has substantial amounts of vitamin A (retinol equivalent) 653.0 µg/100 g, vitamin E (tocopherol equivalent) 2.2 mg/100 g, between 183 and 248 mg/100 g cholesterol, and minor amounts of calcium, phosphorus, vitamin K 60 µg/100 g, vitamin D 1.2 µg/100 g and also a low protein content [3]. Additionally, it is a well-known fact that butter’s color is given by the presence of carotene (lycopene), vitamin A, and other fat-soluble pigments [7].

Regarding the color differences of butter from different species, Milovanovic (2020) found that butter produced from goat’s milk is lighter than butter produced from cow’s milk. The values of the red–green color parameter (a*) are very close to each other, and butter produced from cow’s milk is more yellowish [8].

In terms of chemical composition, the lipid fraction of cow’s milk is dominated by triacylglycerols (98%) and small quantities of monoacylglycerols and diacylglycerols, glycolipids, ether lipids free fatty acids, phospholipids, and sterols [4,9]. According to the Codex Alimentarius 2018, butter is a fatty product made only from milk and/or products obtained from milk, containing a minimum of 80% milkfat, a maximum of 16% water, and a maximum of 2% non-fat solids [10]. In the European Union, the unsalted butter must contain 82% *m*/*m* milk fat [11]. Nowadays, besides traditional butter (80% *m*/*m* milk fat), other butter products such as reduced-fat or low-fat butter have been produced. The Codex Alimentarius [12] regulates those products as products exclusively obtained from milk with a milk fat content between 10% and 80%.

Butter has a unique texture and flavor; generally, lipids have a desirable impact on many food products’ sensory properties by affecting the mouth-feel, color, texture, and rheological properties. The composition of fatty acids determines the physical characteristics (such as hardness), stability, the nutritional value of lipids, and also of butter. All-natural lipids consist of saturated fatty acids, monoenoic fatty acids, polyunsaturated fatty acids in different concentrations and differ in detailed fatty acids composition [13,14]. Butter with more than 80% *m*/*m* milk fat was analyzed in various studies about cow feed system variation [15], the seasonal variation of fatty acids [16], rheological properties [17]. However, to the author’s knowledge, there are no other studies on butter with different fat content less than 80%. Besides hardness, plasticity, and viscosity [18], another texture parameter with particular importance for butter is adhesiveness; and consequently, the objective of the current study was to investigate the textural parameters such as adhesiveness and viscosity using mechanical textural techniques; also, the outside and inside color parameters were measured in accordance with the chemical composition of the produced butter samples. From the perspective of chemical composition, the samples were analyzed in terms of fat content, moisture, non-fat solids, and also the fatty acids composition of butter samples was determined using a GC-MS.

## 2. Materials and Methods

This study’s experimental material consisted of butter with different fat contents, from 43% to over 80% (Table 1). For butters samples preparation, the 30% fat pasteurized cream (from cow’s milk) was purchased from a local producer (Suceava, Romania), and the butter samples were produced through the churning process at room temperature (20 °C). The cream temperature was between 10–12 °C. The obtained butter samples were packed in closed plastic containers, and a part was stored in refrigerated conditions (10 ± 2 °C), and a part was stored in freezing conditions (−20 ± 2 °C) until further analysis.

### 2.1. Physicochemical Analysis

The fat content of the butter samples was measured as described in ISO 3727-3:2003 [19], the non-fat solids content was measured according to ISO 3727-2:2003 [20], and the water content was also determined by heating the sample (about 5 g) at 102 ± 2 °C in accordance with ISO 3727-1:2003 [21]. The physicochemical analysis was performed after 24 h of refrigeration, and the results were expressed as percentages.

Butter color measurement. For color evaluation, the Konica Minolta CR-400 ChromaMeter (Konica Minolta, Tokyo, Japan) was used, and the color parameters such as brightness (L*), red–green color parameter (a*), yellow–blue color parameter (b*) were measured applying the Commission Internationale de l’Eclairage (CIE) L*a*b* uniform color space assay [22].

The color of butter samples was measured both outside and inside (by cutting the sample), and based on the measured color parameters it was calculated the tone or hue angle (h^0^), whiteness index (WI), color intensity or chroma (C*), yellowness index (YI), and as follows [8,23]:

h^0^ = tan^−1^ (b*/a*) (1)

(2) $$\mathrm{WI}\;=\;100-\sqrt{{\left(100-L*\right)}^{2}+{a*}^{2}+{b*}^{2}}$$

(3) $$C*\;=\;\sqrt{{a*}^{2}+{b*}^{2}}$$

YI = 142.86 · b*/L*(4)

ΔE* = [(ΔL*)^2^ + (Δa*)^2^ + (Δb*)^2^]^1/2^ (5)

### 2.2. Fatty Acid Analysis

The butter samples stored in freezing conditions for less than a month were used for fat extraction at 50 °C according to ISO 14156:2001 [24,25]. The preparation of fatty acid methyl esters involved the fat solubilization in n-hexane and the utilization of 0.2 mL potassium hydroxide (2 mol/L KOH) methanolic solution as a transesterification reagent, following the method described by ISO 15884:2002 [26]. The composition of butter’ fatty acids methyl esters was determined by gas chromatography-mass spectrometry (GC-MSQP 2010 Plus, Shimadzu, Kyoto, Japan) equipped with an AOC-01 auto-injector, and using a SUPELCOWAX^TM^10 capillary column (60 m length, 0.25 mm in diameter, with 0.25 µm film thickness, Supelco Inc., Bellefonte, PA, USA). The carrier gas was helium with a flow rate and a split ratio of 0.8 mL/min and 1:24, respectively. The injection volume was set at 1 µL. Each measurement was made in triplicate. The GC-MS operated through the procedure described by Oroian, 2020 [27]. A 37 component FAME Mix standard (FAME Mix, Restek, Bellefonte, PA, USA, 35077) was used, and the identification of butter fatty acids methyl esters was made by comparing their retention times with those of the FAME mix standard. Furthermore, the resulting mass spectra were confronted to the ones from the GC-MS database (NIST MSSearch 2.0). The quantification process of the fatty acids involved measuring the area of the peaks and the use of these values in the calculation of the fatty acid concentration.

### 2.3. Texture Parameter Analysis

For textural analysis, a Mark 10-ESM 301 (Mark 10 Corporation, Copiague, NY, USA) texture analyzer was used. The texture parameters measured were viscosity (V) and adhesiveness (A), using three different probes: 120° conical probe—PC120, 5 mm diameter spherical probe—PS5, and 10 mm diameter spherical probe—PS10. The testing speed was set at 10 mm/min, and the trigger force was set at 0.05 N. During the testing of butter samples, the force (N) evolution versus penetration depth (mm) was recorded by the MESUREgauge software at a reading rate of 5 points per second (pps). The penetration depth was set to 5 mm for PC120 and PS10 and 2.5 mm for PS5. The samples were analyzed at 10 °C. Adhesiveness was calculated as the negative force–penetration depth area (A, N·mm) [28], while viscosity was quantified as the negative peak force (V, N), [29,30].

All reagents used for analysis were of analytical grade (Sigma Aldrich, Darmstadt, Germany).

### 2.4. Statistical Analysis

The obtained results were used for the analysis of variance (ANOVA), performed with STATGRAPHICS CENTURION XVI software (Trial Version), while the Unscrambler 9.7 software (Camo, Oslo, Norway) was used for Principal Component Analysis (PCA). The Pearson correlation was also made by SPSS 13.0 software (SPSS Inc. Chicago, IL, USA). The results are expressed as means of three measurements.

## 3. Results and Discussion

### 3.1. Physicochemical and Color Evaluation

As shown in Table 1, the manufactured butter samples (marked from 1 to 5) presented a fat content ranging between 43.95% and 84.60%, a moisture content ranging from 12.58% to 55.14%, and between 0.72% and 1.42% non-fat solids. The variation of fat in butter samples was achieved by eliminating different amounts of buttermilk.

The non-fat solids determination implied the water evaporation from a known mass of butter and fat extraction with petroleum. The results were in the same range as those reported by Queirós, 2016 [7].

For color evaluation, the Commission Internationale de l’Eclairage—CIE L*a*b* assay was applied; the color parameters (brightness—L*, red—a*, green—−a*, yellow—b*, blue—−b*, tone—h^0^, whiteness index—WI, color intensity—C*, yellowness index—YI) and color differences (ΔE*) of butter samples being shown in Table 2 and Table 3. From Table 2, it can be observed that butter’s average brightness values varied from 90.46 to 94.87, the outside and inside measurements being close to each other. The ANOVA statistical analysis divided the brightness results obtained for inside and outside measurements into four statistical groups (A–D), results of outside measurements belonging to the same statistical group with inside measurements (^C^). The red–green (a*) color parameter is in the negative region with average values between –7.36 and –6.41, more toward green, while the yellow–blue color parameter (b*) presented positive values greater than 18.84, more toward yellow. In contrast to the other two-color parameters (L* and a*), the outside b* color parameter of butter samples showed greater values than the inside ones, indicating that the butter color is yellower on the outside after 24 h of production, the ANOVA statistics highlighted this difference at a level of *p* < 0.001. In a study on the influence of caw feed on the color of butter, it was also observed that L* and a* values do not show significant variations; however, relevant differences were detected for the b* color parameter [31].

For butter color, b* is an important parameter that differentiates the analyzed sample in seven statistical groups (^A–G^), and according to O’Callaghan’s 2016 study, the yellowness b* color parameter was directly correlated with trans-β-carotene content [31]. The yellowness color of milk fat is due to vitamin A, carotenes, and other pigments [32].

In terms of color intensity—C* (or colorfulness), the mean values varied from 19.92 to 33.69; the highest value was recorded to the outside of butter samples with the highest fat content—^aA^ ANOVA statistical group—(no.5 samples, 84.6% fat), while the lowest value has been registered by the inside color measurement of the butter samples with the lowest fat content—^eH^ ANOVA statistical group—(no.1 samples, 43.95% fat). The tone-h^0^, express as a sexagesimal degree from 0 to 360, defines the proportion of the dominant spectral component, such as red (0° or 360°), yellow (90°), green (180°), or blue (270°) [33,34].

The h^0^ values of analyzed butter samples were higher than 101.24 and lower than 110.28, showing a yellow dominant spectral component with some degree of green color and presenting variations with the samples’ chemical composition.

In addition to the previously mentioned, the whiteness index (WI) and the yellowness index (YI) were also calculated. It can be observed that 87% fat butter (samples no.5) had the highest yellowness index both inside (49.23 ^aB^) and outside (51.21 ^aA^) and the lowest whiteness index (outside 65.40 and inside 66.65). Furthermore, the whiteness index’s highest value was recorded by the samples of butter with the lowest fat content (samples no.1, 43.95% fat). This similarity was also confirmed by the ANOVA statistical analysis (*p* < 0.001).

An essential and valuable parameter to assess food color variations is represented by the color difference (ΔE*); in this study, this color parameter was calculated to estimate the difference in the inside and outside color of butter samples. In Table 3, the color differences for all analyzed samples are shown and can be observed a significant difference between inside, outside color measurement, and between butter samples. The color differences results varied from 1.23 to 14.31; the smallest color differences were recorded between the outside and inside measurements of the same samples (1.25, 1.32 and 1.57). In contrast, the highest color differences were recorded between the butter samples’ outside color measurements with the highest fat content and the inside color measurement of the one with the lowest fat content (13.05, 13.89 and 10.51). Moreover, the color difference between dairy samples can be easily perceived by the human eye if the color difference value is greater than 3 (ΔE* > 3), if ΔE* < 1.5, the color differences could not be perceived by the human eye, and if 1.5 < ΔE* < 3 minor color differences could be distinguished by the human eye [23,35]. Analyzing the color difference results smaller than 3 shows that both inside-inside and outside-outside color measurements are representative in differentiating butter samples with variation in chemical composition; only one value of ΔE* was less than 3. The color difference results were also statistically analyzed; ANOVA highlighted a significant difference at a level of *p* < 0.05 between the produced butter samples.

### 3.2. Fatty Acid Analysis

The means fatty acids concentration of butter samples’ fat phase, expressed as percentages, are presented in Table 4, and from 37 fatty acids examined, only 18 were quantified. The identified fatty acids were divided according to chain length and depending on the degree of unsaturation into four categories, and as can be seen, the long-chain saturated fatty acids represent a significant part of the total composition, ranging between 46.911% and 56.966%. The monounsaturated fatty acids are also an important category of the chemical composition, with an average value of 25.482%.

The short and medium-chain saturated fatty acids (C4:0–C13:0) represent an essential category of milk fat substances given that these fatty acids have a high digestibility, are entirely used as an energy source by the human body, and do not cause obesity [36].

In addition to the previously mentioned, some short and medium-chain saturated fatty acids (C4:0, C8:0, C12) have been reported to possess antiviral, antifungal, [37] and antibacterial activity (against Gram-positive bacteria especially) in animal and in vitro studies [36,38].

The fatty acids of analyzed butterfat are approximate: 69.12% saturated, 25.482% monounsaturated, and 5.301% polyunsaturated; the obtained values of fatty acids’ concentration being similar to the concentration of fatty acids reported by Rutkowska, 2011 [36] and correspondingly with those reported by Staniewski, 2021 [39]. The polyunsaturated and saturated fatty acids ratio represents an important index used to evaluate the impact of diet on human health. In the case of butter, this index value is about 0.076, while the unsaturated and saturated fatty acids ratio is 0.445, which are smaller than World Health Organization recommendations [40] but close to those for dairy products [41].

The fatty acids’ concentration of butter samples is shown in Table 5, and it can be observed that the most abundant saturated fatty acid in analyzed butter samples was palmitic acid (36.917–74.692 μg/mg), followed by stearic (14.984–30.317 μg/mg), and lauric (6.613–13.381 μg/mg), while the most abundant monounsaturated fatty acid was oleic (23.255–47.051 μg/mg). Only linoleic (5.180–8.352 μg/mg) and linolenic (0.730–1.306 μg/mg) acids were identified from the polyunsaturated fatty acids category. These two fatty acids take part in biological processes and are considered essential and must be provided by the diet because the body can not synthesize them [42]. As expected, the category with the highest fat content (samples no. 5) also showed the highest values of fatty acids’ concentration from analyzed butter samples. Furthermore, 15 of 18 fatty acids showed significant differences (ANOVA) at a level of *p* < 0.05 between the produced butter samples. Taking into account the high content of palmitic and lauric acids, which are considered pro-atherogenic, and the small amounts of unsaturated fatty acids (UFA) of analyzed butter samples, the index of atherogenicity (IA) was calculated (IA = (C12:0 + (4·C14:0) + C16:0)/UFA) and ranged between 1.789 for no. 1, 2 and 3 samples to 1.850 for the 4 and 5 samples [43].

The atherogenic index represents a more complex indicator than the ratio of polyunsaturated and saturated fatty acids, which is too general and inappropriate for evaluating the atherogenicity of foods. According to Yurchenko (2018), the consumption of food products with a small value of atherogenic index can decrease blood total cholesterol levels [44]. The index of atherogenicity has been used widely by many researchers for the characterization of red, green, and brown seaweeds (0.20–3.50) [45,46], of meat and meat products (0.16–0.80) [47,48], and also of dairy products (1.5–4), [49].

In addition to the fatty acids discussed in some samples, traces of arachidic acid (C20:0) were also identified.

### 3.3. Textural Evaluation

**Figure 1:**
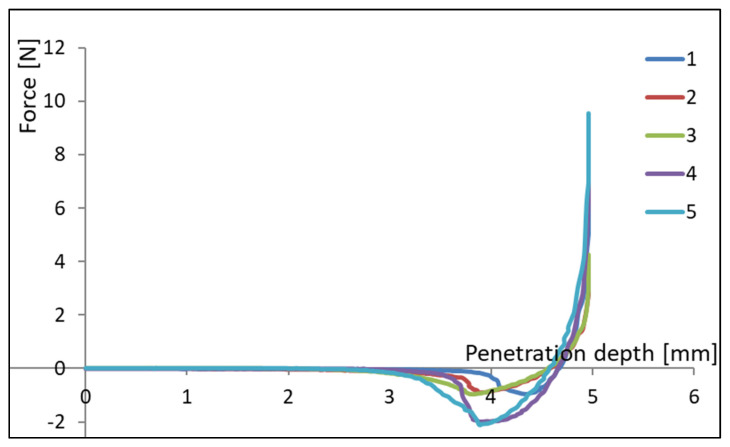
The PS_10_ penetrometer force–penetration depth curves of butter samples.

Besides plasticity, which is the property that controls spreadability, another important texture parameter of lipids is represented by viscosity or consistency [18]. In Table 6 the measured values of viscosity and adhesiveness are shown with three different penetrometers (PS10, PC120, and PS5). The recording of the test curves in force versus penetration depth coordinates (Figure 1) allowed the calculation of the adhesiveness as energy (joules) or work (N·m), similar to the definition of Bourne, 2002 [50] and ISO 11036:2020 [51]. The viscosity ranged between 0.24 and 2.12 N, while the butter samples’ adhesiveness ranged between 0.286 N·mm to 18.19 N·mm (or mJ). Regarding the used penetrometers, the PC_120_ presented the highest values on both viscosity and adhesiveness measurements, whereas the PS_5_ measurements registered the smallest values.

A positive Pearson correlation was observed between fat content, viscosity, and adhesiveness measured with PS_10_ and PS_5_ penetrometers (*p* < 0.05), while the moisture content negatively influences these texture parameters (*p* < 0.05). Besides the influence they have on the nutritional value and sensory properties, [52] fatty acid composition also influences butter’s textural characteristics; the high content of saturated fatty acids also causes a high hardness and low spreadability at refrigeration temperature [53].

Furthermore, in a study by Staniewski 2021 about butter firmness, it was observed that the increase of C14:0 and a decrease of C18:1 also enlarged the butter firmness [39].

**Figure 2:**
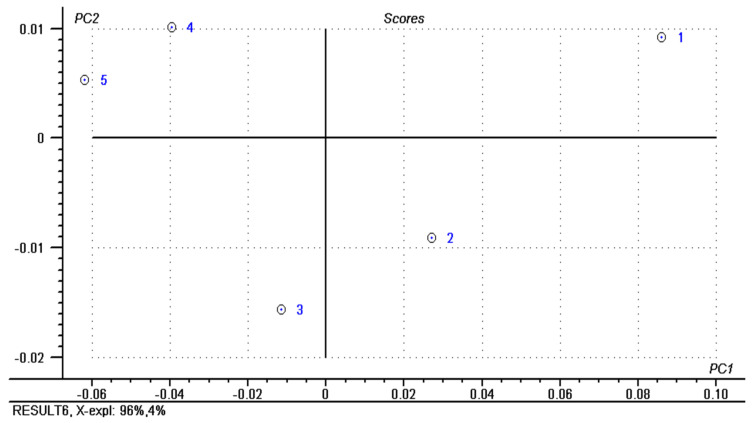
PCA scores of butter samples.

**Figure 3:**
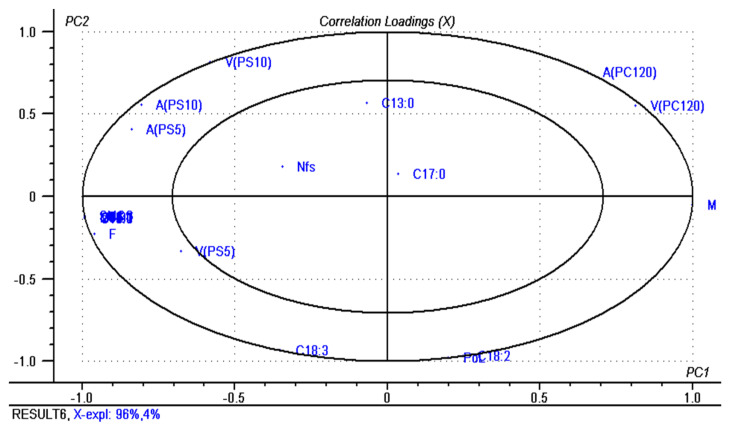
PCA loadings of butter samples.

PCA (principal component analysis) was also performed on physicochemical parameters, fatty acids composition, and texture parameters of analyzed butter samples. Those two principal components (PC1 and PC2) describe all data variation (100%); the first component—PC1, explains 96% while the second component—PC2, explains 4%. The principal component analysis results are represented by Scores (Figure 2) and Correlations Loadings (Figure 3); the objective is to highlight the important information from the measured data and to reduce the number of the variables. Based on the PCA Scores, it can be noticed that the analyzed butter samples are divided into different quadrants according to chemical composition. The central parameters of correlations loadings matrix (heptadecanoic, tridecanoic fatty acid, and non-fat solids) have an unimportant effect in samples differentiation, whereas those from the outside (fat, moisture, viscosity, adhesiveness, butyric, caproic, caprylic, capric, linoleic, linolenic acids) present a strong influence in butter differentiation. It can be seen from the Scores that the first component (PC1) separates the no. 3, 4, and 5 butter samples from the no.1 and 2 butter samples based on PS adhesiveness, fatty acids concentration, and fat content, which are in contrast with water content (Correlation Loadings). Additionally, from the Correlation Loadings, it can be observed that viscosity and adhesiveness measured with PS penetrometers significantly influence the 4 and 5 butter samples projection; the PC_120_ viscosity and adhesiveness influence the no.1 butter samples projection, while the projection of the no. 3 butter sample is influenced by the content of linolenic acid (C18:3). The maximum value reached in describing the data variation highlights the usefulness of principal component analysis in butter classification using texture parameters and chemical composition.

## 4. Conclusions

The study carried out on different butter samples highlighted that fat content strongly influences only the b* color parameter; it was also observed that L* and a* values do not show significant variations with chemical composition. For butter color, the yellowness b* color parameter is a relevant indicator that differentiated the analyzed samples into seven statistical groups (^A–G^). Furthermore, the whiteness index’s highest value was recorded by the butter samples with the lowest fat content, and this similarity is also confirmed by the ANOVA statistical analysis (*p* < 0.001). The main saturated fatty acid contained in the butter samples was palmitic acid (36.917–74.692 μg/mg), followed by stearic (14.984–30.317 μg/mg) and lauric (6.613–13.381 μg/mg); the most abundant monounsaturated fatty acid was oleic (23.255–47.051 μg/mg), while from the polyunsaturated fatty acids category, only linoleic (5.180–8.352 μg/mg) and linolenic (0.730–1.306 μg/mg) acids were identified. Regarding the texture parameters, it was observed that viscosity and adhesiveness measured with PS_10_ and PS_5_ penetrometers were positively influenced by fat content, while the moisture content negatively influences these texture parameters.

## Figures and Tables

**Table 1:** Physicochemical parameters of butter samples express as mean (SD).

Sample	Fat (%)	Moisture (%)	Non-Fat Solids (%)
1	43.95 (0.34)	55.14 (0.68)	0.78 (0.14)
2	55.42 (0.82)	43.52 (0.87)	0.72 (0.29)
3	65.72 (0.22)	33.26 (0.56)	0.98 (0.31)
4	77.12 (0.33)	21.87 (0.41)	0.91 (0.23)
5	84.60 (0.92)	12.58 (0.48)	1.42 (0.12)

**Table 2:** Butter color parameters.

Sample	Outside Color Parameters—Mean (SD)						
	L*	a *	b*	C	h^0^	YI	WI
1	93.72 ^bB^ (0.36)	−7.11 ^cF^ (0.04)	19.25 ^eG^ (0.07)	20.52 ^eG^ (0.09)	110.28 ^aA^ (0.05)	29.34 ^eH^ (0.01)	78.53 ^aA^ (0.02)
2	91.70 ^cC^ (0.65)	−6.68 ^bD^ (0.03)	25.78 ^cD^ (0.44)	26.63 ^cD^ (0.42)	104.53 ^dF^ (0.31)	40.16 ^cE^ (0.40)	72.09 ^cE^ (0.21)
3	94.85 ^aA^ (0.17)	−6.62 ^abCD^ (0.02)	24.44 ^dE^ (0.050	25.32 ^dE^ (0.04)	105.16 ^bD^ (0.08)	36.80 ^dF^ (0.15)	74.16 ^bD^ (0.08)
4	90.53 ^dD^ (0.06)	−7.29 ^dE^ (0.08)	27.44 ^bC^ (0.92)	28.39 ^bC^ (0.91)	104.88 ^cE^ (0.31)	43.30 ^bC^ (1.49)	70.06 ^dG^ (0.89)
5	92.19 ^cC^ (0.45)	−6.57 ^aBC^ (0.09)	33.05 ^aA^ (0.17)	33.69 ^aA^ (0.18)	101.24 ^eG^ (0.10)	51.21 ^aA^ (0.01)	65.40 ^eI^ (0.07)
F-ratio	109.60	148.88	683.60	645.47	1440.10	807.29	834.36
*p*-value	*p* < 0.001	*p* < 0.001	*p* < 0.001	*p* < 0.001	*p* < 0.001	*p* < 0.001	*p* < 0.001
	inside color parameters—mean (SD)						
1	90.46 ^dD^ (1.36)	−6.48 ^aAB^ (0.10)	18.84 ^eG^ (0.75)	19.92 ^eH^ (0.74)	109.00 ^aB^ (0.41)	29.74 ^eH^ (0.73)	77.88 ^aB^ (0.08)
2	94.87 ^aA^ (0.39)	−6.94 ^cE^ (0.01)	22.89 ^dF^ (0.29)	23.91 ^dF^ (0.28)	106.86 ^bC^ (0.20)	34.47 ^dG^ (0.59)	75.53 ^bC^ (0.36)
3	93.53 ^bB^ (1.55)	−6.61 ^bCD^ (0.14)	24.36 ^cE^ (0.33)	25.24 ^cE^ (0.36)	105.19 ^cD^ (0.12)	37.20 ^cF^ (0.10)	73.91 ^cD^ (0.03)
4	91.74 ^cC^ (0.08)	−7.36 ^dE^ (0.04)	27.20 ^bC^ (0.02)	28.18 ^bC^ (0.01)	105.13 ^cDE^ (0.09)	42.36 ^bD^ (0.07)	70.63 ^dF^ (0.03)
5	92.30 ^cC^ (0.12)	−6.41 ^aA^ (0.02)	31.81 ^aB^ (0.26)	32.44 ^aB^ (0.25)	101.39 ^dG^ (0.14)	49.23 ^aB^ (0.48)	66.65 ^eH^ (0.27)
F-ratio	19.17	127.76	841.83	792.73	907.51	1483.95	2667.73
*p*-value	*p* < 0.001	*p* < 0.001	*p* < 0.001	*p* < 0.001	*p* < 0.001	*p* < 0.001	*p* < 0.001
F_out-ins_-ratio	5.04	123.16	676.81	639.56	1026.97	921.27	1084.46
*p*_out-ins_-value	*p* < 0.01	*p* < 0.001	*p* < 0.001	*p* < 0.001	*p* < 0.001	*p* < 0.001	*p* < 0.001

Different lowercase letters (a–d) in a column show significant differences between the outside or inside groups (*p* < 0.05)—ANOVA test. Different uppercase letters (A–I) in a column show significant differences between the outside and inside groups (*p* < 0.05)—ANOVA test.

**Table 3:** The color differences of butter samples.

Sample		Outside					Inside				
		1	2	3	4	5	1	2	3	4	5
1	outside	-	7.23	5.71	9.16	13.89	3.35	4.19	5.53	8.58	13.05
2			-	3.43	2.11	7.28	6.56	4.30	2.32	1.57	6.06
3				-	5.30	9.01	6.73	1.58	1.32	4.23	7.80
4					-	5.88	8.14	6.29	4.35	1.23	4.79
5						-	14.31	10.51	8.79	5.91	1.25
1	inside						-	5.67	5.88	8.01	12.60
2								-	2.01	5.34	9.29
3									-	3.44	7.55
4										-	4.73
5											-

**Table 4:** The fatty acids composition (%) of fat butter samples.

Fatty Acid Concentration (%)								
	Name	Abbreviation	RT ± 0.5 min	Min	Max	Mean	SD	Total
Short and middle-chain saturated	Butyric	C4:0	6.078	0.831	1.589	1.340	0.131	16.782
	Caproic	C6:0	7.910	1.657	3.183	2.600	0.609	
	Caprylic	C8:0	10.824	1.301	2.367	2.037	0.424	
	Capric	C10:0	14.251	3.206	5.512	4.772	0.950	
	Lauric	C12:0	17.639	3.703	7.412	5.832	1.350	
	Tridecanoic	C13:0	19.241	0.147	0.239	0.201	0.036	
Long-chain saturated	Myristic	C14:0	20.488	3.020	4.956	4.022	0.780	52.338
	Pentadecanoic	C15:0	22.310	1.389	1.967	1.705	0.254	
	Palmitic	C16:0	23.961	29.761	34.767	32.592	1.888	
	Heptadecanoic	C17:0	25.773	0.419	1.078	0.770	0.272	
	Stearic	C18:0	27.919	12.322	14.198	13.249	0.708	
Monounsaturated	Myristoleic	C14:1	21.427	1.318	1.877	1.659	0.217	25.482
	cis-10-pentadecenoic	C15:1	23.002	0.072	1.386	0.499	0.146	
	Palmitoleic	C16:1	24.518	2.178	2.861	2.593	0.283	
	cis-10 Heptadecanoic	C17:1	26.438	0.000	0.094	0.040	0.009	
	Oleic	C18:1cis (n9)	28.563	18.890	23.494	20.691	1.872	
Polyunsaturated	Linoleic	C18:2 cis (n6)	29.854	2.959	7.629	4.661	1.875	5.301
	Linolenic	C18:3 (n3)	31.840	0.000	1.008	0.640	0.050	

**Table 5:** Fatty acids concentration (μg/mg) of butter samples.

Name	Abbreviation	Butter Samples					Anova
		1	2	3	4	5	
Butyric	C4:0	1.528 ^c^	1.954 ^bc^	2.309 ^abc^	2.736 ^ab^	3.091 ^a^	**
Caproic	C6:0	2.963 ^c^	3.790 ^bc^	4.479 ^abc^	5.306 ^ab^	5.995 ^a^	*
Caprylic	C8:0	2.313 ^d^	2.959 ^cd^	3.497 ^bc^	4.143 ^ab^	4.681 ^a^	**
Capric	C10:0	5.430 ^d^	6.946 ^cd^	8.209 ^bc^	9.724 ^ab^	10.987 ^a^	**
Lauric	C12:0	6.613 ^d^	8.459 ^cd^	9.997 ^bc^	11.843 ^ab^	13.381 ^a^	**
Tridecanoic	C13:0	0.289 ^cd^	0.226 ^d^	0.342 ^bc^	0.405 ^ab^	0.458 ^a^	**
Short- and middle-chain saturated		19.136	24.334	28.833	34.157	38.593	**
Myristic	C14:0	4.590 ^c^	5.871 ^b^	6.938 ^b^	8.219 ^a^	9.287 ^a^	***
Pentadecanoic	C15:0	1.926 ^d^	2.464 ^cd^	2.912 ^bc^	3.449 ^ab^	3.897 ^a^	***
Palmitic	C16:0	36.917 ^c^	47.219 ^b^	55.804 ^b^	66.107 ^a^	74.692 ^a^	***
Heptadecanoic	C17:0	0.993 ^a^	0.707 ^a^	1.230 ^a^	1.050 ^a^	1.663 ^a^	NS
Stearic	C18:0	14.984 ^c^	19.166 ^b^	22.651 ^b^	26.833 ^a^	30.317 ^a^	***
Long-chain saturated		59.41	75.427	89.535	105.658	119.856	**
Myristoleic	C14:1	1.892 ^d^	2.420 ^cd^	2.861 ^bc^	3.389 ^ab^	3.829 ^a^	***
cis-10-pentadecenoic	C15:1	0.544 ^a^	0.695 ^a^	0.822 ^a^	0.973 ^a^	1.100 ^a^	NS
Palmitoleic	C16:1	2.945 ^d^	3.767 ^cd^	4.451 ^bc^	5.273 ^ab^	5.958 ^a^	***
cis-10 Heptadecanoic	C17:1	0.043 ^a^	0.055 ^a^	0.065 ^a^	0.077 ^a^	0.087 ^a^	NS
Oleic	C18:1cis (n9)	23.255 ^c^	29.745 ^b^	35.153 ^b^	41.643 ^a^	47.051 ^a^	***
Monounsaturated		28.679	36.682	43.352	51.355	58.025	**
Linoleic	C18:2 cis (n6)	5.180 ^a^	6.625 ^a^	7.830 ^a^	7.239 ^a^	8.352 ^a^	NS
Linolenic	C18:3 (n3)	0.730 ^a^	0.934 ^a^	1.104 ^a^	1.143 ^a^	1.306 ^a^	NS
Polyunsaturated		5.910	7.560	8.934	8.382	9.658	NS

NS—not significant (*p* > 0.05), * *p* < 0.05, ** *p* < 0.01, *** *p* < 0.001; Different lowercase letters. in a row show significant differences between the groups (*p* < 0.05).

**Table 6:** Texture parameters (mean–SD) of analyzed butter.

Probe	PS_10_		PC_120_		PS_5_	
Sample	Viscosity	Adhesiveness	Viscosity	Adhesiveness	Viscosity	Adhesiveness
1	0.96 (0.02)	6.364 (0.49)	1.78 (0.11)	18.19 (0.68)	0.24 (0.01)	0.286 (0.08)
2	0.92 (0.06)	7.98 (0.25)	1.28 (0.07)	10.5 (0.26)	0.28 (0.01)	0.316 (0.08)
3	0.98 (0.04)	8.38 (0.21)	1.18 (0.03)	6.73 (0.19)	0.44 (0.02)	0.462 (0.10)
4	1.98 (0.10)	14.31 (0.39)	1.64 (0.14)	17.71 (0.67)	0.46 (0.01)	0.772 (0.18)
5	2.12 (0.07)	15.68 (0.26)	1.74 (0.05)	14.61 (0.72)	0.48 (0.01)	1.197 (0.16)

PS_10_—10 mm diameter spherical probe, PC_120_—120° conical probe, PS_5_—5 mm diameter spherical probe.
